# Biohybrid motor neuron spheroid composed of graphene/HUVEC/neural cell for 3D biosensing system to evaluate drug of amyotrophic lateral sclerosis

**DOI:** 10.1186/s40580-025-00495-0

**Published:** 2025-06-26

**Authors:** Minkyu Shin, Taehyeong Ha, Sangeun Lee, Chenzhong Li, Jin-Ha Choi, Jeong-Woo Choi

**Affiliations:** 1 Department of Chemical & Biomolecular Engineering, Sogang University, Seoul, 04170 Republic of Korea; 2 Department of Chemistry and Chemical Biology, Rutgers, The State University of New Jersey, 123 Bevier Road, Piscataway, NJ 08854 USA; 3 Biomedical Engineering, School of Medicine, The Chinese University of Hong Kong, Shenzhen, 518172 China; 4 School of Chemical Engineering, Jeonbuk National University, 567 Baekje-daero, Deokjin-gu, Jeonju-si, Jeollabuk-do 54896 Republic of Korea

**Keywords:** Biohybrid motor neuron spheroid, Biohybrid brain organoid, Reduced graphene oxide nanoparticles, Human umbilical vein endothelial cells, 3D biosensing system, Amyotrophic lateral sclerosis drug evaluation

## Abstract

A 3D motor neuron (MN) spheroid has been developed to investigate neurodegenerative and neuromuscular junction (NMJ) disease. However, core necrosis and reduced neurogenesis, impairing neural network formation, were observed as the MN spheroid matured. In this study, to enhance neural network formation, a biohybrid MN spheroid composed of neural cells/reduced graphene oxide (rGO)/human umbilical vein endothelial cells (HUVECs) was generated for the first time and applied to 3D biosensing system for MNJ disease. By incorporating rGO and HUVECs at the onset of human neural stem cell (hNSC) culture, rGO and HUVECs were evenly distributed within MN spheroid generated by differentiation of hNSC, which improved oxygen- and nutrient- supply by reduction of core necrosis, and enhanced neurogenesis. The fabricated biohybrid MN spheroid improved neural network formation and electrophysiological signal. This method was also applied to generate biohybrid cerebral organoids from human induced pluripotent stem cells (hiPSCs), emphasizing its versatility for diverse 3D neural models. Then, a 3D NMJ biosensing system was fabricated by positioning the biohybrid MN spheroid with muscle bundles to evaluate its utility in neuromuscular disease modeling. Biohybrid MN spheroids generated from induced pluripotent stem cells of sporadic amyotrophic lateral sclerosis (ALS) patients were used to make NMJ. Reduced contraction of the connected muscle bundle due to ALS could be restored by upon treatment with the bosutinib, ALS drug, demonstrating the potential use for drug screening. The method to generate biohybrid spheroid can be applied to generation of various biohybrid brain organoids, and the proposed 3D NMJ biosensing system can be used to drug screening of diverse neuromuscular diseases.

**Supplementary Information:**

The online version contains supplementary material available at 10.1186/s40580-025-00495-0.

## Graphical abstract

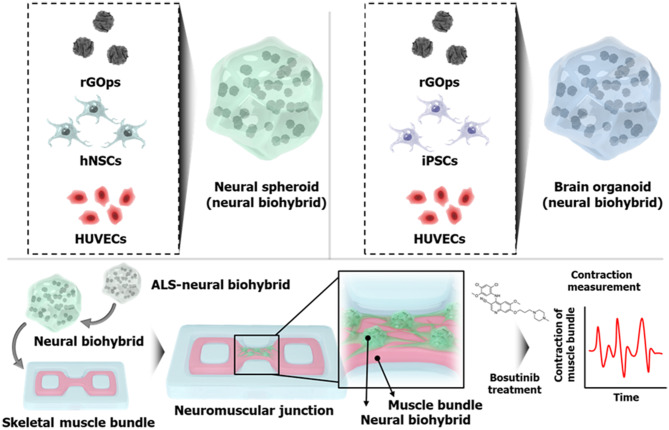

## Introduction

Bioengineering neural cell cultures in vitro have recently attracted significant attention for modeling degenerative diseases and drug evaluation [1, 2]. Most neurodegenerative diseases like Alzheimer’s, Parkinson’s, and amyotrophic lateral sclerosis (ALS) remain challenging to treat due to our limited understanding of motor and cognitive function complexities [2, 3]. Numerous studies have attempted to fabricate neural interconnections, network formations, and functions for development of degenerative disease models for effective treatment [4–6]. Neuromuscular junction (NMJ) disease is a degenerative and debilitating neurological disorder that is difficult to treat, primarily due to nerve damage and the inability to form NMJs [7]. ALS, commonly called Lou Gehrig’s disease, is an NMJ disorder caused by motor neuron (MN) degeneration and death [8, 9]. Riluzole and edaravone, modern drugs approved for ALS treatment, only prolong life by 2–3 months or slow the disease’s progression, but there are no disease models for the evaluation of ALS drug effects [10, 11]. Therefore, new and reliable platforms are urgently needed to verify the effects of the drugs.

Three-dimensional (3D) cultures using human neural stem cells (hNSCs) and induced pluripotent stem cells (iPSCs) have been utilized to mimic developmental and degenerative diseases [12–14]. Contrasting two-dimensional (2D) cell cultures, 3D cell cultures, including spheroids and organoids, using hNSCs and human iPSCs indicate the high similarity with the function and structure of human organs [12]. Accordingly, 3D cell culture models have been actively applied not only in disease modeling but also in the field of neural and tissue regeneration. In particular, the ability of the 3D neural models to generate intrinsic electrophysiological signals makes them well-suited for studying neural connectivity and responses in a physiologically relevant context [15]. Despite these advantages, current spheroid and organoid models often face necrotic core during long-term culture in vitro due to the lack of microvasculature and limited oxygen, nutrient, and growth factor diffusion. These insufficient cell-ECM interactions affect inefficient neurogenesis and unwanted gliogenesis inside the aggregated spheroid and organoid [16, 17]. These factors reduce the neural network, neurogenesis, and neuroplasticity, complicating accurate drug assessments in degenerative disease models. A promising approach to address these challenges involves the development of a synergistic strategy aimed at generating stem cell spheroids and organoids from hybrids, incorporating 3D cell-cell and cell-matrix interactions. As vascular cells within neurons directly support efficient oxygen and nutrient distribution, recent attempts have included vascular structures in 3D models [18–20]. Thus, vascular cells positively affect neuronal proliferation and neurogenesis, potentially serving as a physical track for axon growth [18, 19]. Endothelial cells secrete factors, such as glial-derived neurotrophic factor (GDNF), to enhance neuronal survival and axonal growth [20, 21]. Furthermore, there has been growing interest in 3D biosensing platforms that utilize organoids, spheroids, and other 3D cell culture models [22–25]. Compared to traditional 2D systems, these 3D models more accurately recapitulate the in vivo microenvironment, including cellular architecture, signaling dynamics, and physiological responses. Such structural and functional complexity enhances their relevance as sensing platforms, particularly for monitoring real-time electrophysiological activity, drug responses, and disease progression. These advantages significantly increase their potential for application in advanced biosensing systems, bridging the gap between in vitro assays and clinical relevance.

Graphene is one of the most widely used materials in various studies due to its outstanding properties [26, 27]. Due to these unique properties, graphene has achieved tremendous attention as one of the most promising materials for biomedical applications. In particular, from a biological perspective, graphene exhibits exceptional bioactivity by directly influencing cellular behavior, including promoting neuronal adhesion, proliferation, and differentiation, often without the need for external chemical inducers [28]. Its ability to create a highly favorable microenvironment for cells distinguishes it from conventional biomaterials. Furthermore, graphene-based materials have been implemented as scaffolding platforms to not only physically support cell growth but also to actively enhance neurogenesis, guide neural differentiation, and stimulate human neural stem cell (hNSC) regeneration. These effects arise from graphene’s unique ability to modulate electrical signaling and provide bioelectrical cues critical for neural network formation. As a result, graphene has expanded its utility into diverse applications such as tissue engineering, drug delivery, and regenerative medicine [28–30]. Despite these promising results, the application of graphene in complex 3D biological systems such as spheroid- and organoid-based hybrid models remains underexplored, representing a significant opportunity for advancing neural tissue engineering.

**Fig. 1:**
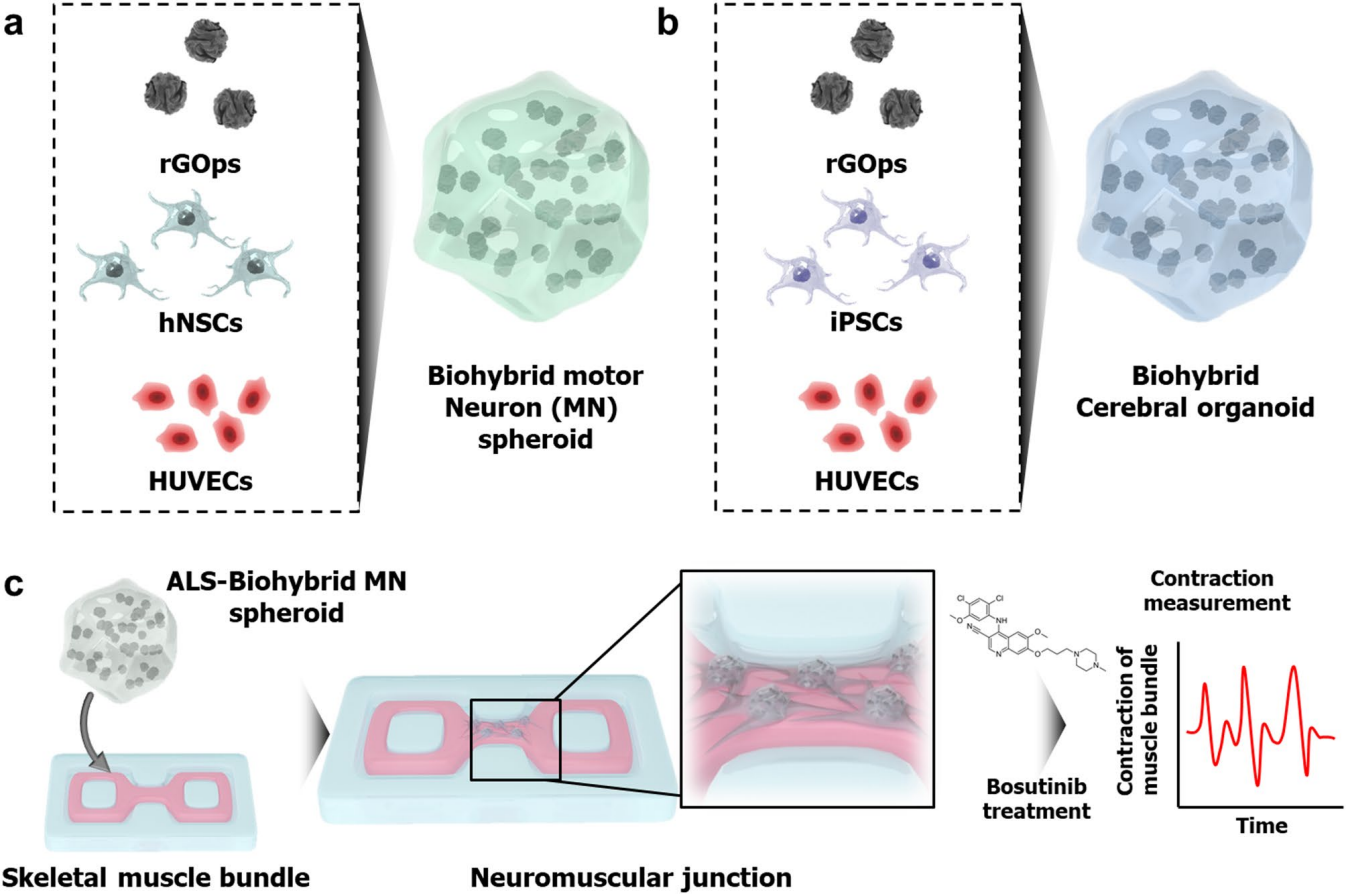
Schematic diagram illustrating the neural biohybrid fabrication strategy and its application to neuromuscular junction formation for drug evaluation of ALS. Schematic diagram illustrating 3D neural spheroid or brain organoid composed of rGO, HUVECs and (**a**) hNSCs or (**b**) IPSCs. (**c**) Schematic illustrating the application of neural hybrids to neuromuscular junction formation for drug evaluation in ALS

Recently, we reported on the development of nano-biohybrid hydrogels composed of COOH-carbon nanotubes (CNT), extracellular matrix (ECM), and motor neuron (MN) spheroids [31]. These materials were designed to enhance the neuromuscular junction between the MN spheroid and muscle bundle. Our study confirmed that the alignment of CNT supports the effective differentiation of MN spheroids and the formation of neuromuscular junctions. However, we still have not resolved the issue of core necrosis in 3D cell structures, such as spheroids. Based on our previous study, in this study, we propose a general method for generating biohybrid MN spheroid or biohybrid cerebral organoid by incorporating the reduced graphene oxide nanoparticles (rGOps) and Human Umbilical Vein Endothelial Cells (HUVECs) into hNSCs or iPSCs for the first time (Fig. 1a and b). The incorporation of rGO at the early stage of hNSC culture played a key role in enhancing electrical conductivity and cellular microenvironments, while co-cultured HUVECs promoted vascular-like networks, leading to improved oxygen and nutrient delivery. As a result, the biohybrid MN spheroids exhibited significantly reduced core necrosis and enhanced neurogenesis, thereby facilitating robust neural network formation and improved electrophysiological activity. This method is not limited to spheroids but is also applicable to other 3D neural models. For example, we successfully generated a biohybrid cerebral organoid by hiPSCs, suggesting broad utility across various 3D neural models. The proposed biohybrid MN spheroid was also applied to the NMJ for ALS drug evaluation (Fig. 1c). The fabricated biohybrid MN spheroids enhanced neurogenesis and differentiation, amplifying NMJ formation in muscle fibers. In addition, NMJ with ALS patient-derived biohybrid MN spheroid (ALS-biohybrid MN spheroid) was fabricated and applied to assess ALS drug efficacy. Notably, the reduced muscle contraction observed in NMJ with the biohybrid was recovered through bosutinib, an ALS drug, indicating a successful drug evaluation. These results substantiate that the proposed biohybrid MN spheroid can be broadly applied as a biosensing system for several NMJ-related diseases, including ALS. By incorporating 2D materials, such as graphene and HUVECs, into hNSCs or iPSCs to generate biohybrid MN spheroid, we provide a general approach for 3D neural biohybrid generation, including neural spheroids or brain organoids that enhance differentiation and neurogenesis.

## Methods/Experimental

### Synthesizing reduced graphene oxide nanoparticles (rGOps)

Graphene oxide (GO) was prepared by oxidating graphite powder (Alfa Aesar, 99.99% purity) using the modified Hummers’ method. Reduced graphene oxide nanoparticles (rGOps) were fabricated from GO colloids (0.5 wt%) through aerosol spray drying. GO colloids were applied through a two-fluid nozzle, with the droplets subsequently dried in a preheated chamber. The flow rate of the carrier gas was measured at 10 L/min, while the precursor feed rate was established at 4.5 mL/min. Additionally, the temperature within the chamber was maintained at 200 °C. Subsequently, rGOps were collected in a sampler.

### 3D biohybrid MN spheroid fabrication

Human neural stem cells (hNSCs) from Takara Bio Inc. (San Jose, USA) and neural stem cells derived from induced pluripotent stem cells (ALS-iPSC) from a 55-year-old Caucasian female ALS patient (iXCells Biotechnologies, San Diego, USA) were cultured on laminin-coated plates. The culture medium consisted of KnockOut™ DMEM/F-12 basal medium (Gibco, ThermoFisher, Waltham, USA) supplemented with StemPro™ NSC SFM Supplement (Gibco), along with 20 ng/mL FGFb and 20 ng/mL EGF (both from Peprotech, ThermoFisher, Waltham, USA). 3D biohybrids MN were formed using round-bottom ultra-low attachment 96-well plates by seeding hNSCs (7.0 × 10^4^ cells per well) with HUVECs at a 7.0 × 10^3^ cells/well density and 0.1 mg/mL rGOps. ALS-iPSC-derived NSCs were generated using the same procedure. Following a duration of 48 h, the culture medium was substituted with a 1:1 combination of EGM-2 (Lonza, Basel, Switzerland) and StemPro hESC medium, which contained 8 ng/mL bFGF, 200 ng/mL Sonic hedgehog (Peprotech), 10 ng/mL activin A (Peprotech), and 50 µM retinoic acid (Sigma-Aldrich, St. Louis, USA) to facilitate biohybrid differentiation. After 20 days, the culture medium was replaced with a 1:1 mixture of EGM-2 (Lonza) and StemPro hESC medium with 10 ng/mL brain-derived neurotrophic factors (BDNF; Peprotech), 10 ng/mL, and glial cell-line derived neurotrophic factors (GDNF; Peprotech) for biohybrid maturation and further cultured for eight days.

### Fabrication of 3D NMJ using neural biohybrids and muscle bundles

After pre-formation and differentiation of muscle bundle composed of skeletal muscle cell and ECM through the polymer mold, differentiated 3D biohybrid MN spheroid were positioned on the muscle bundle using the ECM proteins (Matrigel 30%, 4 mg/mL fibronectin, 0.5 U/mg thrombin) to polymerize and spread into muscle bundles for co-culture. BDNF and GDNF, both at 10 ng/mL, were added to the co-culture medium to support neural biohybrid viability and muscle differentiation.

### Measurement of muscle bundle contractions by the drug treatment

Glutamate (100 µM) was administered into the co-culture medium to evaluate excitatory neurotransmitter activity, and muscle contractions were verified using an optical microscope. Furthermore, bosutinib (100 µM) was introduced into the co-culture medium to assess the efficacy of this ALS drug candidate, with subsequent muscle contractions confirmed on Days 1, 3, and 7, also through optical microscopic examination. The analysis of muscle bundle contractions was conducted utilizing ImageJ software.

## Results and discussion

### Synthesized rGOps analysis

**Fig. 2:**
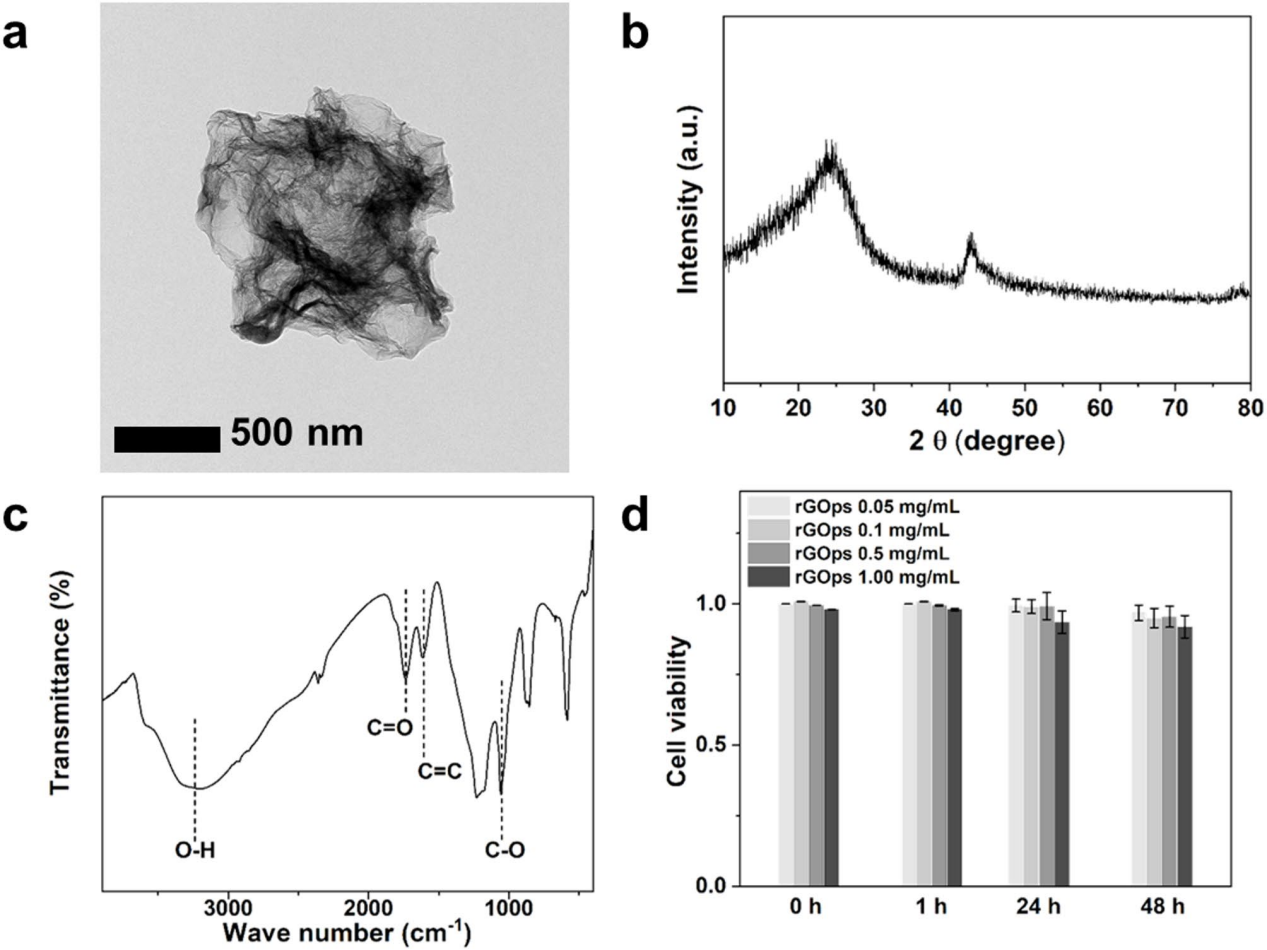
Confirmation and characterization of rGOps synthesis. (**a**) TEM image of the rGOps. (**b**) XRD pattern analysis of rGOps. (**c**) FT-IR spectra for rGOps within the 4000–400 cm⁻¹ range. (**d**) Cell viability assay of rGOps

Neural differentiation, maturation, and enhanced neurogenesis of the biohybrid MN spheroid are paramount for stabilizing and enhancing neural network formation. As previously mentioned, graphene promotes neurogenesis, neuronal differentiation, and neural stem cell regeneration. In addition, graphene and graphene oxide (GO) are broadly incorporated into biological systems due to their excellent biocompatibility and physical and electrical properties [32, 33]. Notably, 3D structures with low aggregation properties can further enhance graphene’s excellent intrinsic properties compared to 2D structures with high aggregation properties [34]. Therefore, rGOps in this study were prepared with aerosol spray drying and GO colloids. Figure 2a includes an HR-TEM image of the prepared rGOps, confirming that the particle was approximately 600 nm. Next, as shown in Fig. 2b, XRD pattern analysis indicated that rGOps exhibited broad diffraction peaks at 23–25.5°. In addition, some functional groups were observed in Fig. 2c, including -OH (a peak ~ 3400 cm^− 1^), C = O (1720 cm^− 1^), C = C (1635 cm^− 1^), and C-O (1080 cm^− 1^) [35, 36].

Cell viability of rGO to hNSC for generation of biohybrid MN spheroid was evaluated through an MTT assay incubated with different rGOps concentrations for 1 h, 24 h, and 48 h (Fig. 2d). A statistically significant difference was found from the control group at a 1.0 mg/mL concentration, whereas a nominal difference was observed at 0.5 mg/mL and below. However, aggregated spheroids were not formed at a 0.5 mg/mL concentration, and the cells tended to unwind; thus, 0.1 mg/mL was determined as the ideal concentration for entering the spheroids (Fig. S1). From these results, we confirmed that the rGOps were successfully synthesized. Moreover, the optimized concentration of rGOps did not interfere with the spheroid formation of hNSCs, indicating that they can be stably utilized in the fabrication of biohybrid MN spheroids.

### Generating rGOps and HUVECs-incorporated biohy-brid MN spheroids

**Fig. 3:**
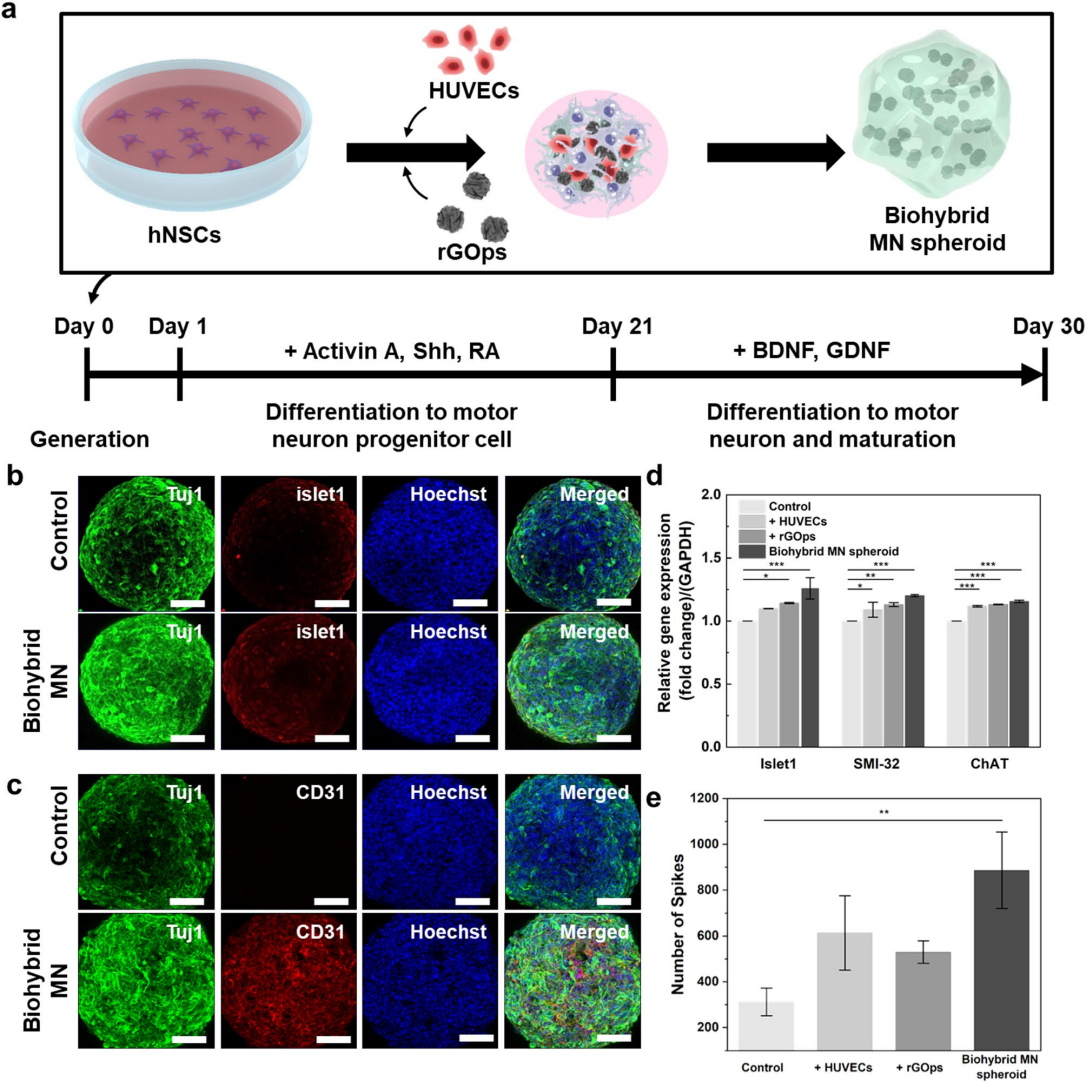
Generation of Biohybrid MN spheroid composed of neural cells, rGOps, and HUVECs. (**a**) Schematic diagram depicting the biohybrid MN spheroid containing neural cells, rGOps, and HUVECs. Immunostaining images of representative marker expression in differentiated control spheroids and biohybrid MN spheroids using Tuj1 (growth neurite, green), Hoechst 33,342 (nuclear, blue), (**b**) islet1 (differentiation MN marker, red), and (**c**) CD31 (vascular differentiation marker, red). Scale bar: 100 μm. (**d**) Biohybrid MN spheroid qPCR analysis. Normalized islet1, ChAT, and SMI-32 (differentiation makers) expression levels in the biohybrid MN spheroid and control spheroids (control, control with rGOps, and control with HUVECs). *P < 0.05, **P < 0.01, and ***P < 0.001. Error bars indicate the standard error of the mean for four measurements. (**e**) Electrophysiological activity analysis of biohybrid MN spheroids and control spheroids on Day 35 of differentiation. *P < 0.05, **P < 0.01, and ***P < 0.001. Error bars correspond to the standard error of the mean from three measurements

3D neural spheroids evidenced improvement in differentiation and maturation compared to 2D cultures [12, 37]. hNSCs or iPSCs, 0.1 mg/mL rGOps, and HUVECs were seeded into a round-bottom 96-well plate to generate spheroids (Fig. 3a). The generated spheroid differentiated and matured into biohybrid MN spheroids, as mentioned in previous studies [12].

In addition, a standard MN marker, islet1, expressed more biohybrid MN spheroid than the control spheroids (Fig. 3b). Moreover, class II beta-tubulin (Tuj1) immunostaining, a typical neuron marker, confirmed extended and tangled neurites on Day 28 of differentiation compared to control spheroids (Fig. 3b). As depicted in Fig. 3c, hNSCs and HUVECs were uniformly mixed and distributed in the formed biohybrid MN spheroid, whereas a blood vessel-like structure is not apparent in the control spheroid. Furthermore, MN differentiation markers, islet1, SMI-32, and ChAT gene expressions were measured through a qPCR analysis (Fig. 3d). SMI-32, islet1, and ChAT expressions increased relative to their control spheroid (control, control with rGOps, and control with HUVECs). Graphene expressed high biocompatibility and electrical conductivity in supporting cell adhesion, inducing synapse formation, and increasing nerve growth [38, 39]. Differentiated biohybrid MN spheroid and their relative control spheroids were also evaluated through electrophysiological signal analysis to detect neural signals. To confirm the effectiveness of rGOps and HUVECs for enhancement of electrophysiology, such as action potentials and functional activation, spontaneous activation was observed through a microelectrode array (MEA). As shown in Fig. 3e, the number of spikes of the biohybrid MN spheroid indicated 886.67 ± 111.2, which is 2.84, 1.45, and 1.67 times higher than that of the control, without HUVECs, and without rGOps, respectively.

**Fig. 4:**
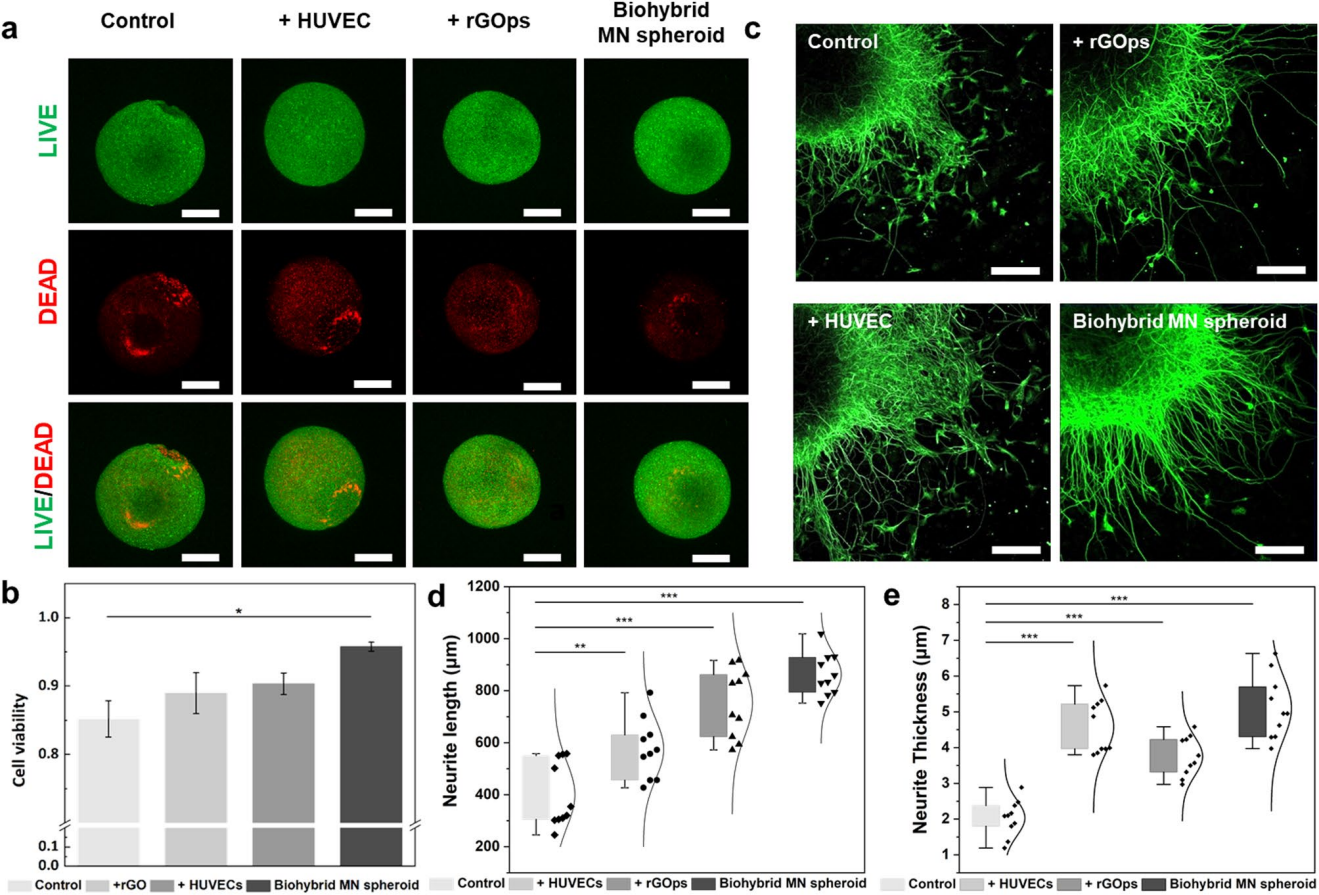
Characterization of biohybrid MN spheroid. (**a**) Live (green)/dead (red) cell viability assay of the biohybrid MN spheroid and control spheroids (control, control with rGOps, and control with HUVECs). Scale bar: 200 μm. (**b**) Cell viability assay through Live/Dead staining. *P < 0.05. Error bars correspond to the standard error of the mean from seven measurements. (**c**) Immunostaining images of the biohybrid MN spheroid and control spheroids with *Tuj1* (green). Scale bar: 100 μm. Neurite length (**d**) and (**e**) thickness measurements for the biohybrid MN spheroids and control spheroids on Day 35 of differentiation. *P < 0.05, **P < 0.01, and ***P < 0.001. Error bars correspond to the standard error of the mean from ten measurements

In addition to the enhancement of the electrophysiology of the spheroid, rGOps, and HUVEC affect cell viability. For this, we confirmed spheroid cell viability on Day 7 using Live/Dead staining. As shown in Fig. 4a, the dead cell (red) of the biohybrid MN spheroid was significantly lower than that of MN spheroids of hNSCs only, control with HUVEC, and control with rGOps. In addition, the cell viability of biohybrid MN spheroid was confirmed for approximately 95.76% of biohybrid MN spheroid and 85.2%, 88.96%, 90.33% of control spheroids (control, control with rGOps, and control with HUVECs), respectively (Fig. 4b). Endothelial cells secrete factors, such as GDNF, to enhance neuronal survival and axon growth, and internalized graphene-based nanoparticles interact with intracellular organelles to influence NSC proliferation and differentiation [21, 28]. Regarding neurite outgrowth, the biohybrid motor neuron MN spheroids exhibited significantly enhanced neurite length and thickness compared to the control group. Specifically, the average neurite length increased from 400.43 ± 39.6 μm in the control group to 575.31 ± 36.48 μm with the addition of HUVECs and 754.01 ± 41.6 μm with rGOps. Notably, the incorporation of both components in the biohybrid MN spheroids resulted in the longest neurites, averaging 862.97 ± 25.77 μm, representing more than a 2.1-fold increase compared to the control. Furthermore, in terms of neurite thickness, a similar trend was observed. While control spheroids showed an average neurite thickness of 2.03 ± 0.16 μm, the addition of HUVECs increased this to 4.58 ± 0.23 μm, and rGOps to 3.76 ± 0.17 μm. The biohybrid MN spheroids showed the most substantial enhancement, with an average thickness of 5.11 ± 0.28 μm, more than 2.5 times thicker than the control (Fig. 4c–e). From these results, it was confirmed that the co-introduction of rGOps and HUVECs not only promoted the differentiation of hNSCs into neural cells but also enhanced the generation of electrophysiological signals from the resulting MN spheroids. The presence of rGOps likely contributed to the facilitation of neural maturation, while the incorporation of HUVECs supported the formation of vascular-like structures, which stabilized the internal 3D microenvironment and provided an additional positive effect on cellular viability and function.

### Applying biohybrid MN spheroids to the NMJ formation

**Fig. 5:**
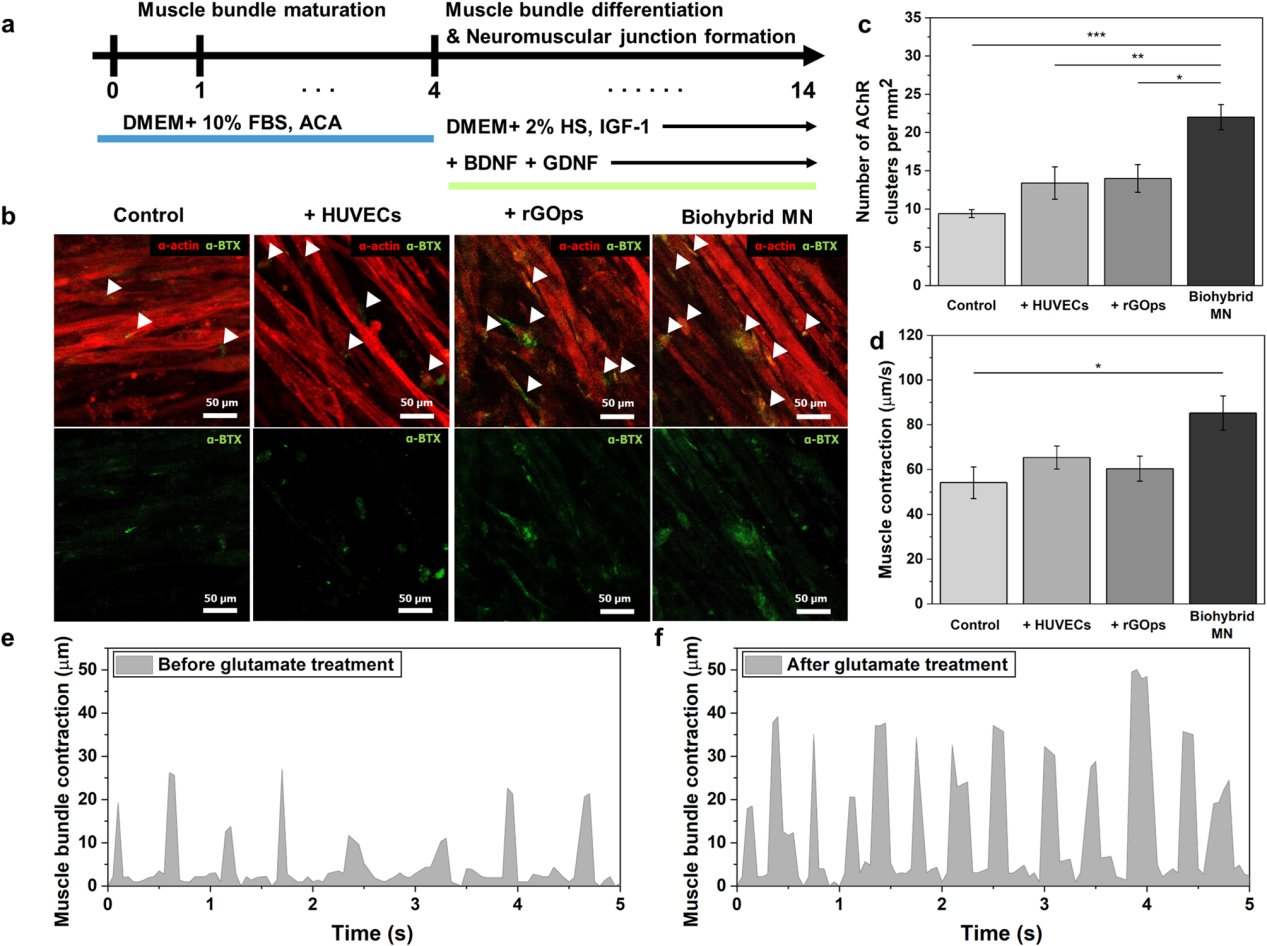
3D NMJ system fabrication and characterization. (**a**) Co-culture preparation and differentiation of muscle bundles and biohybrid MN spheroids. (**b**) Immunostaining images of representative marker expression on Day 14 of NMJ differentiation with biohybrid MN spheroids and control spheroids (control, control with rGOps, and control with HUVECs). α-actinin (sarcomeric α-actinin, red), nAChR (α-BTX, green). Scale bar: 50 μm. White arrows indicate the location of NMJ. (**c**) AChR cluster quantification (per µm^2^). *P < 0.05, **P < 0.01, and ***P < 0.001. Error bars correspond to the standard error of the mean from three measurements. (**d**) Spontaneous contractions in muscle bundles with biohybrid MN spheroids and control spheroids. *P < 0.05. Error bars correspond to the standard error of the mean from three measurements. (**e**) Muscle bundle contractions in the 3D NMJ system with biohybrid MN spheroids before glutamate and (**f**) after 100 µM glutamate treatment

In a previous study, the generation and differentiation of muscle bundles were considered when applying biohybrid MN spheroids to the neuromuscular junction (NMJ), as illustrated in Fig. S2 [31]. Hydrogels composed of muscle cells (C2C12) and extracellular matrix (ECM) were mixed and polymerized within a 3D polymer mold to induce differentiation and maturation of muscle cells. After the differentiation of the muscle bundle for Day 14, immunostaining of myotube sarcomeric α-actinin (α-actinin; green) verified that the generated muscle bundle had indicated aligned myotubes (Fig S3b). In addition, spontaneous muscle contraction was observed at 22.0527 μm/s on Day 7 of differentiation (Fig. S2c and Video S1). For this, CNT-COOH was introduced to induce neurogenesis when mixed with nano-biohybrid hydrogels [40, 41]. Therefore, after obtaining mature myotubes, nano-biohybrid hydrogels mixed in pre-differentiated biohybrid MN spheroids (approximately 10 per muscle bundle), ECM proteins, and CNT-COOH were fabricated and spread on muscle bundles in a 3D mold to create a 3D NMJ system (Fig. S3 and Fig. 5a). In addition, the co-culture medium was replenished with BDNF and GDNF to support biohybrid MN spheroid viability in the muscle bundle differentiation medium. Immunostaining was performed on actin filaments (F-actin; red), α-bungarotoxin (α-BTX; white), and Tuj1 (a neuronal branch/neurite marker; green) to confirm NMJ formation on Day 14 of co-culture differentiation (Fig. S4). NMJs using biohybrid MN spheroids exhibited the neurites evenly connected with the muscle bundle. Furthermore, immunostaining for sarcomeric α-actinin (α-actinin; green) and α-bungarotoxin (α-BTX; green) was conducted on Day 14 of NMJ differentiation for biohybrid MN spheroids and control spheroids. The Acetylcholine receptor (AChR) region marked as ‘α-BTX’ represents the NMJ region.

When comparing control spheroids and biohybrid MN spheroid myotubes, the average number of AChR clusters per µm^2^ was 9.43, 13.29, 15.14, and 21.85 (biohybrid MN spheroid). Comparatively, the number of AChR clusters per µm2 in biohybrid MN spheroids was significantly higher than in control spheroids (Fig. 5b and c). On Day 14 of co-culture, spontaneous muscle bundle contractions averaged 54.11, 65.35, 60.24, and 85.21 μm/s in the 3D NMJ system with control spheroids and biohybrid MN spheroids (Fig. 5d). These muscle bundle contractions were confirmed after adding glutamate to chemical stimulate control spheroids and biohybrid MN spheroids. Glutamate, an excitatory neurotransmitter, is often used to chemically stimulate 3D NMJ systems as it specifically stimulates biohybrid MN spheroids and not muscle fibers. Accordingly, muscle bundle contractions gradually increased when 100 µM of glutamate was treated in the 3D NMJ system with biohybrid MN spheroid. Figure 5e and f show that the muscle bundle contractions following the glutamate treatment enhanced about 1.96 times with biohybrid MN spheroids than the 3D NMJ system with a control spheroid only (Videos S2). These results demonstrate that the rGOps and HUVEC of the biohybrid MN spheroids effectively formed neuromuscular junctions (NMJs) when connected to muscle cells. The neural differentiation promoted by rGOps and HUVECs led to the development of mature neurons, which facilitated more efficient synaptic connectivity with muscle fibers. Furthermore, this enhanced neural-muscle coupling promoted stronger and more synchronized muscle contractions, likely due to improved acetylcholine release at the NMJ and better maintenance of synaptic integrity enabled by vascular support and conductive microenvironments, ultimately resulting in improved movement dynamics.

### 3D ALS-NMJ drug evaluation using an ALS-biohybrid disease model

**Fig. 6:**
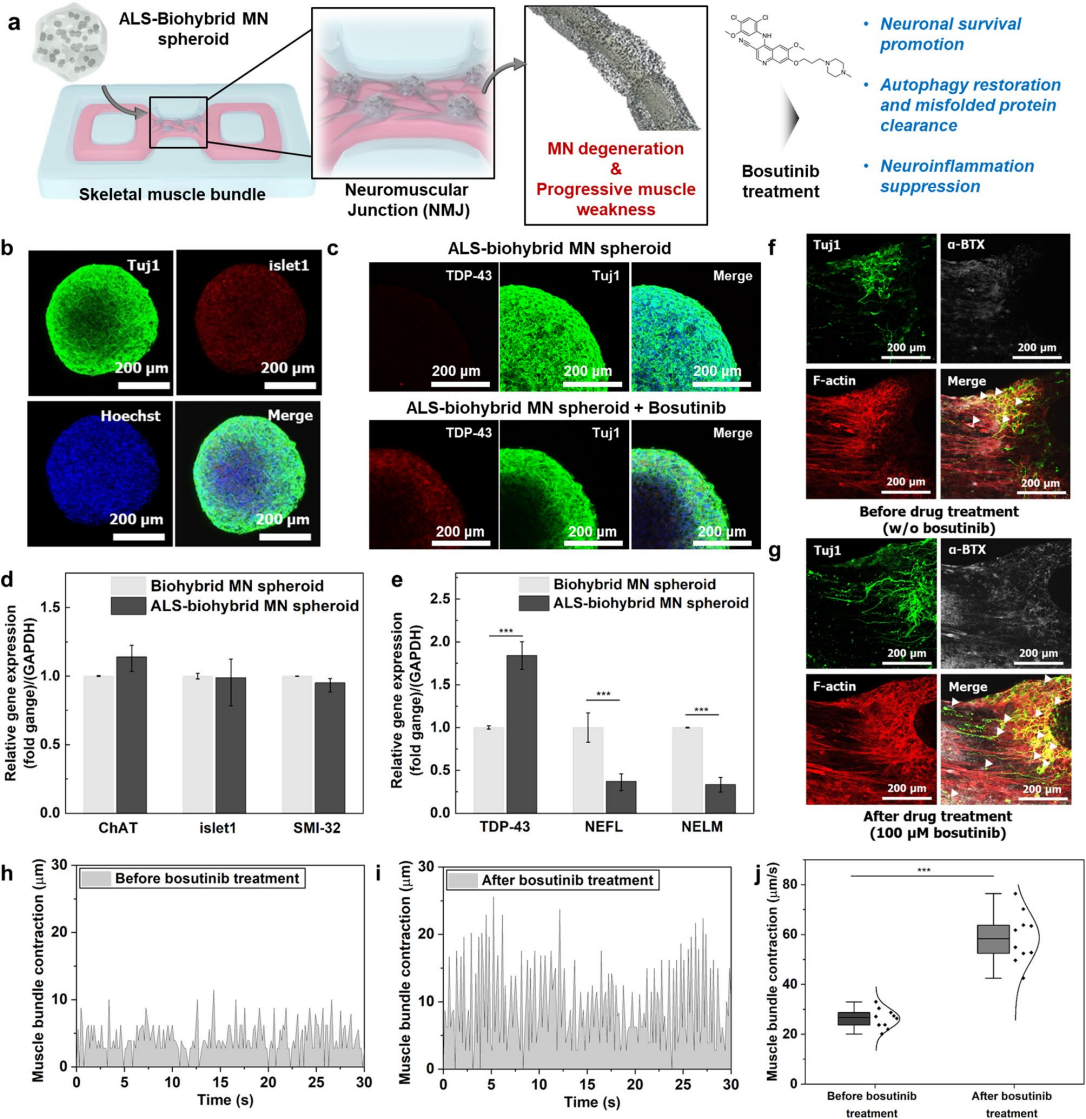
3D NMJ system with ALS-biohybrid MN spheroid fabrication and drug treatment. (**a**) Schematic diagram of the 3D NMJ system for drug evaluation. (**b**) Immunostaining images of representative marker expression on Days 28 of differentiation in the ALS-biohybrid MN spheroids. *Tuj1* (growth neurite, green), *Hoechst 33,342* (nuclear, blue), *islet1* (MN differentiation marker, red). (**c**) Immunostaining images of cytoplasmic *TDP-43* accumulation before and after ALS-biohybrid MN spheroid drug treatment. (**d**) *SMI-32*,* islet1*, and *ChAT* mRNA levels on Day 35 for both the ALS-biohybrid MN spheroids and the biohybrid MN spheroids (control). Error bars show the standard error of the mean from four measurements. (**e**) *TDP-43*,* NEFL*, and *NEFM* mRNA levels on Day 35 for both ALS-biohybrid MN spheroids and biohybrid MN spheroids (control). *P < 0.05, **P < 0.01, and ***P < 0.001. Error bars correspond to the standard error of the mean from four measurements. (**f**) Immunostaining images of representative marker expression before and (**g**) after drug treatment (100 µM bosutinib) on the NMJ with ALS-biohybrid MN spheroids. Muscle bundle (*F-actin*, red), nAChR (*α-BTX*, white), and motor neurons’ neurite growth (*Tuj1*, green). White arrows indicate the location of NMJ. Confirmation of Muscle contractions (**h**) before drug treatment and (**i**) after drug treatment (100 µM bosutinib) using NMJ with ALS-biohybrid MN spheroids. (**j**) Bosutinib-induced muscle bundle movement in NMJ with the ALS-biohybrid MN spheroid. *P < 0.05. Error bars correspond to the standard error of the mean from ten measurements

The remarkable functional performance of our 3D NMJ system with biohybrid MN spheroid prompted us to explore its potential for ALS drug screening (Fig. 6a). To this end, we applied bosutinib, a tyrosine kinase inhibitor known to mitigate ALS-related pathologies, as a candidate therapeutic compound. The restoration of muscle contractility in this system serves as a functional readout, reflecting the recovery of motor neuron integrity and neuromuscular signaling capacity upon drug treatment. For this, we utilized biohybrid MN spheroid derived from human neural stem cells (hNSCs) obtained from iPSCs of sporadic ALS patients, along with 0.1 mg/mL rGOps and HUVECs, to generate an ALS-biohybrid MN spheroid. After 14 days of differentiation, the expression of islet1 in the ALS-biohybrid MN spheroids was comparable to that of conventional healthy hNSCs-derived biohybrid MN spheroids (Biohybrid MN spheroid) (Fig. 6b). Notably, the analysis revealed no significant difference in Islet1, ChAT, and SMI-32 mRNA levels when comparing the ALS-biohybrid MN spheroid to the biohybrid MN spheroid (control) (Fig. 5b). This suggests that the expression of these specific genes related to neuronal function and differentiation remains consistent between the two types of spheroids, indicating that the ALS condition does not appear to alter the levels of these markers in this control. In addition, cytoplasmic TDP-43 accumulation, a well-known ALS characteristic [42], was also observed in this study through immunostaining and qPCR analysis (Fig. 6c-e). Interestingly, the mRNA levels of cytoplasmic TDP-43 were higher in ALS-biohybrid MN spheroids than in biohybrid MN spheroids. On the other hand, the mRNA levels of the neurofilament light (NEFL) and neurofilament medium (NEFM) chains, markers for nerve fibers, were significantly reduced in ALS-biohybrid MN spheroids. This suggests that while the differentiation patterns of ALS-biohybrid MN spheroids were not significantly different from biohybrid MN spheroids, the expression levels of NEFL and NEFM were reduced during ALS-biohybrid MN spheroid differentiation. Notably, a low level of neurofilament growth factors negatively affects the development and formation of nerve fibers and synapses. These growth factors are essential for promoting the growth and connectivity of neurons, and insufficient amounts can lead to impaired neural development and communication within the nervous system.

As previously mentioned, in the case of ALS, impaired muscle movement occurs due to a series of pathological issues, including MN degeneration, disruption of NMJ, accumulation of misfolded proteins such as TDP-43, and neuroinflammation. So, we treated the ALS drug candidate, bosutinib, used to treat chronic myeloid leukemia, to the ALS-biohybrid MN spheroids for five days. Bosutinib promotes autophagy, reduces the misfolded TDP-43 proteins, restores energy homeostasis, and rescues ALS-neuron degeneration. As determined by immunostaining, ALS-biohybrid MN spheroids treated with bosutinib reduced TDP-43 levels (Fig. 6e). Furthermore, we applied ALS-biohybrid MN spheroid to fabricate NMJ formation for ALS drug evaluation. After 14 days of co-culture without bosutinib, insufficient neurite growth was indicated in muscle bundles (Fig. 6f). Alternatively, neurite in the NMJ system with bosutinib indicated enhanced outgrowth compared to the control (without bosutinib) (Fig. 6g). In addition, muscle contraction sharply increased up to five days after bosutinib treatment (Fig. 6h and i). As shown in Fig. 6j, we verified that the contraction of the muscle bundle was restored from 26.26 ± 1.25 μm/s to 58.77 ± 3.22 μm/s when treated with 100 µM of bosutinib. From these results, the ALS-biohybrid MN spheroid was successfully generated and the contraction of NMJ system with the ALS-biohybrid MN spheroid was restored by the treatment of the ALS drug bosutinib.

### Generation and confirmation of biohybrid cerebral organoids

**Fig. 7:**
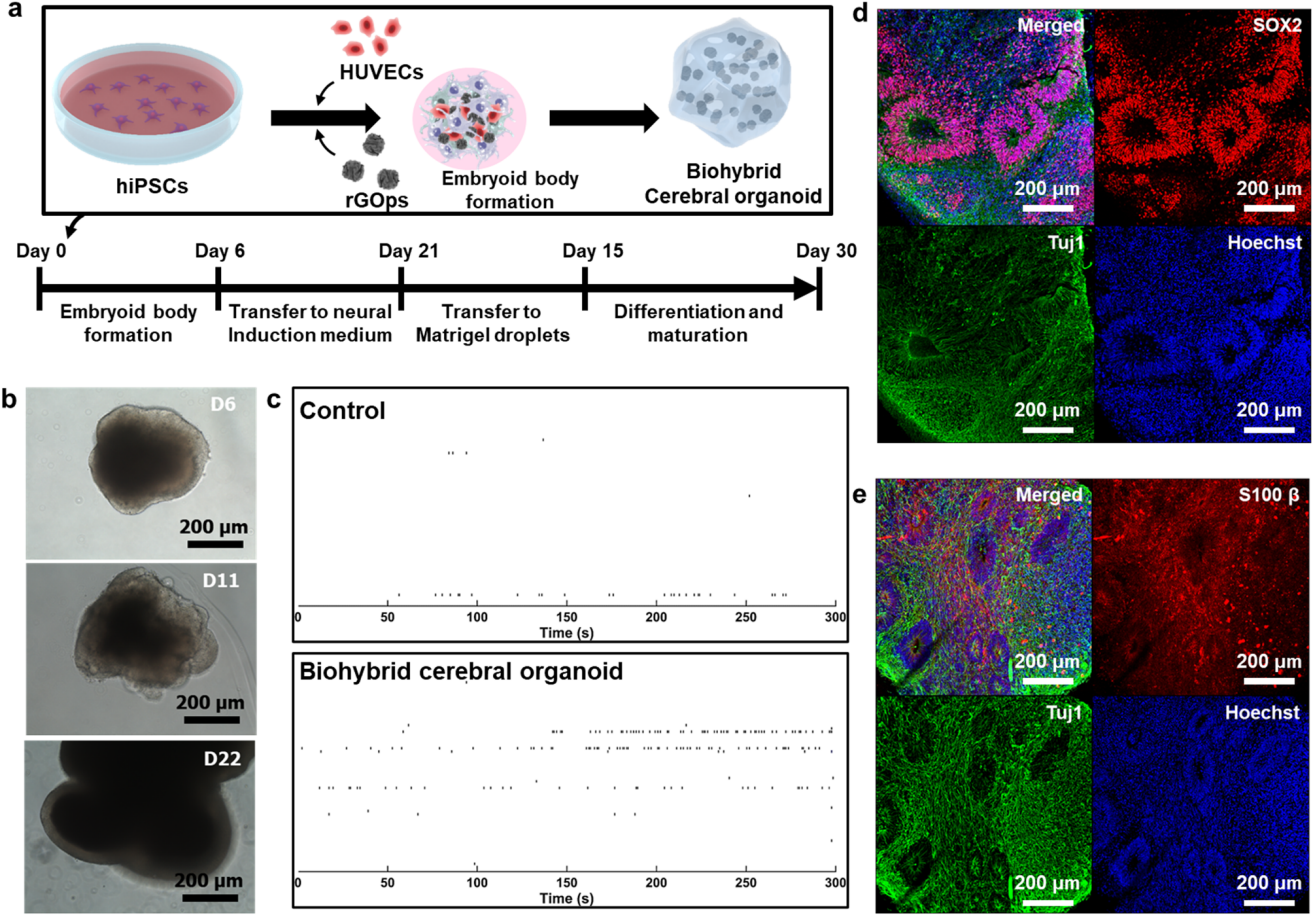
Confirmation of biohybrid cerebral organoid generation using the 3D neural biohybrid method. (**a**) Schematic diagram depicting the generation of the biohybrid cerebral organoid containing rGOps and HUVECs. (**b**) Optical images of the biohybrid cerebral organoid at 6, 11, and 22 days. (**c**) Electrophysiological signal generation of the biohybrid cerebral organoid and control (without the HUVECs and rGOps). (**d**) Immunostaining images of representative marker expression on Day 60 of differentiation for the biohybrid cerebral organoid *Tuj1* (green), *Hoechst 33,342* (blue), *SOX2* (red), and (**e**) *Tuj1* (green), *Hoechst 33,342* (blue), *S100β* (red)

The fabrication method of biohybrid MN spheroids using rGO and HUVECs induced neural differentiation and enhancement of electrophysiological signals of the MN spheroids. We hypothesized that this fabrication method could affect not only the MN spheroids, but also the neurogenesis of brain organoids, which are 3D neural cell structures, and this effect would especially induce the activity of electrophysiological signals in brain organoids. To confirm the use of the generation method for enhanced neural network formation in cerebral organoid, the biohybrid cerebral organoid was generated by the incorporation of rGO/HUVEC into iPSC-derived cerebral organoid during the embryonic body (EB) formation (Fig. 7a). After the neural induction process, the neuroectodermal tissue was indicated on the surface of the EB. Then, the embedding process on day 11 was performed using the Matrigel for the neuroepithelial tissue formation (Fig. 7b). Furthermore, similar to the biohybrid MN spheroid, we analyzed the effects of the rGOps and HUVEC on the generation of the cerebral organoid. As shown in Fig. S5, the cell viability of biohybrid cerebral organoid was confirmed for approximately 95.52% of biohybrid cerebral organoid and 85.84%, 93.42%, 93.92% of control cerebral organoid (control, control with rGOps, and control with HUVECs), respectively (S5). In addition to the cell viability analysis, the MEA analysis was performed to confirm the effects of the rGO/HUVEC on the generation of the biohybrid cerebral organoid. The electrophysiological signal of the generated biohybrid cerebral organoid was verified by the MEA using 90 days of biohybrid cerebral organoid. As shown in Fig. 7c, the 90 days of biohybrid cerebral organoid indicated a higher number of spikes compared to that of without rGO/HUVEC. Similar to the biohybrid MN spheroid, the effect of rGO/HUVECs inside the biohybrid cerebral organoid induced the electrophysiological signal enhancement. Specifically, the electrophysiological signal in the biohybrid cerebral organoid indicated 15.11 ± 2.07µV, representing a higher action potential compared to the control group (13.45 ± 0.84 µV). Additionally, the number of spikes increased from 62 ± 14 to 108.3 ± 47.68 in the biohybrid cerebral organoids (S6). The enhancement of neural differentiation by the rGOps and HUVECs not only increased the frequency of electrophysiological signals but also promoted sufficient maturation of neural cells, leading to the generation of stronger and more defined action potentials.

In particular, the biohybrid cerebral organoids actively showed the formation of neural progenitor cells (SOX2) and vertical structures at the early stage of 30 days, similar to general cerebral organoids (Fig. 7d). In addition, in the case of biohybrid cerebral organoids cultured for more than 90 days, the formation of astrocytes along with neural cells could be confirmed through Tuj1 and s100β (Fig. 7e). Beyond spheroids, we further applied the co-introduction of rGOps and HUVECs to brain organoids and observed comparable enhancements in neural cell formation and electrophysiological signal generation. These results indicate that this approach is not limited to motor neuron spheroids but is broadly applicable to other 3D neural models. Given its effectiveness in promoting neural maturation and functional signal output, this strategy holds strong potential for future applications in a wide range of neural tissue engineering and disease modeling platforms.

## Conclusion

This study proposed a general method for generating biohybrid MN spheroid or cerebral organoids by incorporating HUVECs and rGOps into hNSCs or iPSCs for the first time. The fabricated biohybrid MN spheroid efficiently distributed oxygen and nutrients, directly enhancing stem cell growth, neural network development, neurogenesis, and differentiation. As evidenced, using HUVECs and rGOps for biohybrid MN spheroids directly increases neuronal cell growth and network development inside the biohybrid MN spheroid. Then, the proposed biohybrid MN spheroid was applied for NMJ fabrication. Through immunofluorescence staining, enhanced connectivity between NMJ motor nerve endings and muscle fibers was observed compared to motor neuron spheroids (control). An NMJ with ALS-biohybrid MN spheroid was generated and applied to ALS drug evaluation. The muscle bundle contractions were decreased in the NMJ with ALS-biohybrids and were recovered through bosutinib treatment, an ALS drug. Furthermore, the fabrication method using the rGOps and HUVECs applied to the iPSCs for generation of the biohybrid cerebral organoid. The biohybrid cerebral organoid indicated enhancement of the electrophysiological signal due to the increased neural differentiation by the rGOps and HUVECs. The NMJ with biohybrid MN spheroids evaluated neuromuscular disease drug effects, demonstrating that the proposed biohybrid MN spheroid can be applied to drug screenings and toxicity assessments for diverse neural diseases. Notably, by incorporating 2D materials, such as graphene and HUVECs, into hNSCs or iPSCs to fabricate biohybrid MN spheroids, we devised a general 3D biohybrid structure generation method for MN spheroids or cerebral organoids that can increase differentiation and neurogenesis. The proposed generation method can be used for the generation of various types of 3D cell structures with enhanced differentiation and proliferation for drug screening platforms.

## Electronic supplementary material

Below is the link to the electronic supplementary material.

Supplementary Material 1

Supplementary Material 2

Supplementary Material 3

## Data Availability

The datasets used and/or analyzed during the current study are available from the corresponding author on reasonable request.

## References

[1] T. Parmentier, F.M. James, E. Hewitson, C. Bailey, N. Werry, S.D. Sheridan, R.H. Perlis, M.L. Perreault, L. Gaitero, J. Lalonde, Human cerebral spheroids undergo 4-aminopyridine-induced, activity associated changes in cellular composition and Microrna expression. Sci. Rep. **12**, 9143 (2022). 10.1038/s41598-022-13071-x 35650420 10.1038/s41598-022-13071-x PMC9160269

[2] R. Plen, A. Smith, O. Blum, O. Aloni, U. Locker, Z. Shapira, S. Margel, O. Shefi, Bioengineering 3D neural networks using magnetic manipulations. Adv. Funct. Mater. **32**, 2204925 (2022). 10.1002/adfm.202204925

[3] M. Yan, L. Wang, Y. Wu, L. Wang, Y. Lu, Three-dimensional highly porous hydrogel scaffold for neural circuit dissection and modulation. Acta Biomater. **157**, 252–262 (2023). 10.1016/j.actbio.2022.12.011 36521677 10.1016/j.actbio.2022.12.011

[4] W. Cantley, C. Du, S. Lomoio, T. DePalma, E. Peirent, D. Kleinknecht, M. Hunter, M. Tang-Schomer, G. Tesco, D.L. Kaplan, Functional and sustainable 3D human neural network models from pluripotent stem cells. ACS Biomater. Sci. Eng. **4**, 4278–4288 (2018). 10.1021/acsbiomaterials.8b00622 33304995 10.1021/acsbiomaterials.8b00622 PMC7725274

[5] M.T. Tedesco, D. Di Lisa, P. Massobrio, N. Colistra, M. Pesce, T. Catelani, E. Dellacasa, R. Raiteri, S. Martinoia, L. Pastorino, Soft Chitosan microbeads scaffold for 3D functional neuronal networks. Biomaterials. **156**, 159–171 (2018). 10.1016/j.biomaterials.2017.11.043 29197747 10.1016/j.biomaterials.2017.11.043

[6] J. Yin, A.M. VanDongen, Enhanced neuronal activity and asynchronous calcium transients revealed in a 3D organoid model of alzheimer’s disease. ACS Biomater. Sci. Eng. **7**, 254–264 (2021). 10.1021/acsbiomaterials.0c01583 33347288 10.1021/acsbiomaterials.0c01583

[7] M.L. Liu, T. Zang, C.L. Zhang, Direct lineage reprogramming reveals Disease-Specific phenotypes of motor neurons from human ALS patients. Cell. Rep. **14**, 115–128 (2016). 10.1016/j.celrep.2015.12.018 26725112 10.1016/j.celrep.2015.12.018 PMC4706770

[8] A.M.G. Ragagnin, S. Shadfar, M. Vidal, M.S. Jamali, J.D. Atkin, Motor neuron susceptibility in ALS/FTD. Front. Neurosci. **13**, 532 (2019). 10.3389/fnins.2019.00532 31316328 10.3389/fnins.2019.00532 PMC6610326

[9] H. Lee, J.J. Lee, N.Y. Park, S.K. Dubey, T. Kim, K. Ruan, S.B. Lim, S.H. Park, S. Ha, I. Kovlyagina et al., Multi-omic analysis of selectively vulnerable motor neuron subtypes implicates altered lipid metabolism in ALS. Nat. Neurosci. **24**, 1673–1685 (2021). 10.1038/s41593-021-00944-z 34782793 10.1038/s41593-021-00944-z PMC8639773

[10] M.K. Jaiswal, Riluzole and edaravone: A Tale of two amyotrophic lateral sclerosis drugs. Med. Res. Rev. **39**, 733–748 (2019). 10.1002/med.21528 30101496 10.1002/med.21528

[11] R. de Jongh, X.M. Spijkers, S. Pasteuning-Vuhman, P. Vulto, R.J. Pasterkamp, Neuromuscular junction-on-a-chip: ALS disease modeling and read-out development in microfluidic devices. J. Neurochem. **157**, 393–412 (2021). 10.1111/jnc.15289 33382092 10.1111/jnc.15289

[12] T. Osaki, S.G.M. Uzel, R.D. Kamm, Microphysiological 3D model of amyotrophic lateral sclerosis (ALS) from human iPS-derived muscle cells and optogenetic motor neurons. Sci. Adv **4**, eaat5847 (2018). 10.1126/sciadv.aat5847 10.1126/sciadv.aat5847 PMC6179377 30324134

[13] M. Shin, T. Ha, J. Lim, J. An, G. Beak, J.H. Choi, A.A. Melvin, J. Yoon, J.W. Choi, Human motor System-Based biohybrid Robot-On-a-Chip for drug evaluation of neurodegenerative disease. Adv. Sci. (Weinh). **11**, e2305371 (2024). 10.1002/advs.202305371 38036423 10.1002/advs.202305371 PMC10811491

[14] J.S. Kong, X. Huang, Y.J. Choi, H.G. Yi, J. Kang, S. Kim, J. Kim, H. Lee, Y.A. Rim, J.H. Ju et al., Promoting Long-Term cultivation of motor neurons for 3D neuromuscular junction formation of 3D in vitro using Central-Nervous-Tissue-Derived Bioink. Adv. Healthc. Mater. **10**, e2100581 (2021). 10.1002/adhm.202100581 34363335 10.1002/adhm.202100581

[15] J. An, M. Shin, G. Beak, J. Yoon, S. Kim, H.-Y. Cho, J.-W. Choi, Drug evaluation of parkinson’s disease patient-derived midbrain organoids using mesoporous Au nanodot-patterned 3D concave electrode. ACS Sens. **9**, 3573–3580 (2024). 10.1021/acssensors.4c00476 38954790 10.1021/acssensors.4c00476

[16] C. Rathnam, L. Yang, S. Castro-Pedrido, J. Luo, L. Cai, K.B. Lee, Hybrid SMART spheroids to enhance stem cell therapy for CNS injuries. Sci. Adv. **7**, eabj2281 (2021). 10.1126/sciadv.abj2281 34586845 10.1126/sciadv.abj2281 PMC8480929

[17] M.A. Lancaster, Brain organoids get vascularized. Nat. Biotechnol. **36**, 407–408 (2018). 10.1038/nbt.4133 29734310 10.1038/nbt.4133

[18] L. Song, X. Yuan, Z. Jones, K. Griffin, Y. Zhou, T. Ma, Y. Li, Assembly of human stem Cell-Derived cortical spheroids and vascular spheroids to model 3-D Brain-like tissues. Sci. Rep. **9**, 5977 (2019). 10.1038/s41598-019-42439-9 30979929 10.1038/s41598-019-42439-9 PMC6461701

[19] N. Shin, Y. Kim, J. Ko, S.W. Choi, S. Hyung, S.E. Lee, S. Park, J. Song, N.L. Jeon, K.S. Kang, Vascularization of iNSC spheroid in a 3D spheroid-on-a-chip platform enhances neural maturation. Biotechnol. Bioeng. **119**, 566–574 (2022). 10.1002/bit.27978 34716703 10.1002/bit.27978 PMC9298365

[20] L.A. Rocha, E.D. Gomes, J.L. Afonso, S. Granja, F. Baltazar, N.A. Silva, M.S. Shoichet, R.A. Sousa, D.A. Learmonth, A.J. Salgado, In vitro evaluation of ASCs and HUVECs Co-cultures in 3D biodegradable hydrogels on neurite outgrowth and vascular organization. Front. Cell. Dev. Biol. **8**, 489 (2020). 10.3389/fcell.2020.00489 32612997 10.3389/fcell.2020.00489 PMC7308435

[21] D. Shvartsman, H. Storrie-White, K. Lee, C. Kearney, Y. Brudno, N. Ho, C. Cezar, C. McCann, E. Anderson, J. Koullias et al., Sustained delivery of VEGF maintains innervation and promotes reperfusion in ischemic skeletal muscles via NGF/GDNF signaling. Mol. Ther. **22**, 1243–1253 (2014). 10.1038/mt.2014.76 24769910 10.1038/mt.2014.76 PMC4089004

[22] W. Dou, M. Malhi, Q. Zhao, L. Wang, Z. Huang, J. Law, N. Liu, C.A. Simmons, J.T. Maynes, Y. Sun, Microengineered platforms for characterizing the contractile function of in vitro cardiac models. Microsystems Nanoengineering. **8**, 26 (2022). 10.1038/s41378-021-00344-0 35299653 10.1038/s41378-021-00344-0 PMC8882466

[23] I.M. Gonçalves, R.O. Rodrigues, A.S. Moita, T. Hori, H. Kaji, R.A. Lima, G. Minas, Recent trends of biomaterials and biosensors for organ-on-chip platforms. Bioprinting. **26**, e00202 (2022). 10.1016/j.bprint.2022.e00202

[24] S. Liu, S. Kumari, H. He, P. Mishra, B.N. Singh, D. Singh, S. Liu, P. Srivastava, C. Li, Biosensors integrated 3D organoid/organ-on-a-chip system: A real-time biomechanical, biophysical, and biochemical monitoring and characterization. Biosens. Bioelectron. **231**, 115285 (2023). 10.1016/j.bios.2023.115285 37058958 10.1016/j.bios.2023.115285

[25] Y. Pan, N. Hu, X. Wei, L. Gong, B. Zhang, H. Wan, P. Wang, 3D cell-based biosensor for cell viability and drug assessment by 3D electric cell/matrigel-substrate impedance sensing. Biosens. Bioelectron. **130**, 344–351 (2019). 10.1016/j.bios.2018.09.046 30266425 10.1016/j.bios.2018.09.046

[26] J. Kim, K. Yang, J.S. Lee, Y.H. Hwang, H.J. Park, K.I. Park, D.Y. Lee, S.W. Cho, Enhanced Self-Renewal and accelerated differentiation of human fetal neural stem cells using graphene oxide nanoparticles. Macromol. Biosci. **17**, 1600540 (2017). 10.1002/mabi.201600540 10.1002/mabi.201600540 28394476

[27] Y. Polo, J. Luzuriaga, J. Iturri, I. Irastorza, J.L. Toca-Herrera, G. Ibarretxe, F. Unda, J.R. Sarasua, J.R. Pineda, A. Larranaga, Nanostructured scaffolds based on bioresorbable polymers and graphene oxide induce the aligned migration and accelerate the neuronal differentiation of neural stem cells. Nanomedicine. **31**, 102314 (2021). 10.1016/j.nano.2020.102314 33059092 10.1016/j.nano.2020.102314

[28] R. Li, J.S. Lin, F. Zheng, The design for autologous bone marrow mesenchymal stem cells combined with nano-graphene material in the treatment of neuropathic pain model mice. Mater. Des. **221**, 110954 (2022). 10.1016/j.matdes.2022.110954

[29] K.H. Li, Z.F. Zhang, D.P. Li, W.S. Zhang, X.Q. Yu, W. Liu, C.C. Gong, G. Wei, Z.Q. Su, B. Ultralight, H. Porous, Shape-Adjustable, and biocompatible 3D graphene minerals via incorporation of Self-Assembled peptide nanosheets. Adv. Funct. Mater. **28**, 1801056 (2018). 10.1002/adfm.201801056

[30] A.F. Girao, J. Sousa, A. Dominguez-Bajo, A. Gonzalez-Mayorga, I. Bdikin, E. Pujades-Otero, N. Casan-Pastor, M.J. Hortiguela, G. Otero-Irurueta, A. Completo et al., 3D reduced graphene oxide scaffolds with a combinatorial Fibrous-Porous architecture for neural tissue engineering. ACS Appl. Mater. Interfaces. **12**, 38962–38975 (2020). 10.1021/acsami.0c10599 32805917 10.1021/acsami.0c10599

[31] T. Ha, S. Park, M. Shin, J.Y. Lee, J.H. Choi, J.W. Choi, Biosensing system for drug evaluation of amyotrophic lateral sclerosis based on muscle bundle and nano-biohybrid hydrogel composed of multiple motor neuron spheroids and carbon nanotubes. Chem. Eng. J. **463**, 142284 (2023). 10.1016/j.cej.2023.142284

[32] T. Ha, S.K. Kim, J.W. Choi, H. Chang, H.D. Jang, pH controlled synthesis of porous graphene sphere and application to supercapacitors. Adv. Powder Technol. **30**, 18–22 (2019). 10.1016/j.apt.2018.10.002

[33] M. Mirza-Aghayan, R. Boukherroub, M. Nemati, M. Rahimifard, Graphite oxide mediated oxidative aromatization of 1,4-dihydropyridines into pyridine derivatives. Tetrahedron Lett. **53**, 2473–2475 (2012). 10.1016/j.tetlet.2012.03.026

[34] X.Z. Tang, N. Srikanth, X.Q. Feng, C.K. Chua, K. Zhou, Reduced graphene oxide/silver hybrid with N,N-dimethyl formamide for oxygen reduction reactions and surface enhanced Raman scattering. RSC Adv. **6**, 102519–102527 (2016). 10.1039/C6RA24322C

[35] R.P. Rimington, J.W. Fleming, A.J. Capel, P.C. Wheeler, M.P. Lewis, Bioengineered model of the human motor unit with physiologically functional neuromuscular junctions. Sci. Rep. **11**, 11695 (2021). 10.1038/s41598-021-91203-5 34083648 10.1038/s41598-021-91203-5 PMC8175425

[36] O. Akhavan, E. Ghaderi, E. Abouei, S. Hatamie, E. Ghasemi, Accelerated differentiation of neural stem cells into neurons on ginseng-reduced graphene oxide sheets. Carbon. **66**, 395–406 (2014). 10.1016/j.carbon.2013.09.015

[37] H.P. Bei, Y. Yang, Q. Zhang, Y. Tian, X. Luo, M. Yang, X. Zhao, Graphene-Based nanocomposites for neural tissue engineering. Molecules. **24**, 658 (2019). 10.3390/molecules24040658 30781759 10.3390/molecules24040658 PMC6413135

[38] D. Kim, M. Shin, J.H. Choi, J.W. Choi, Actuation-Augmented biohybrid robot by hyaluronic Acid-Modified Au nanoparticles in muscle bundles to evaluate drug effects. ACS Sens. **7**, 740–747 (2022). 10.1021/acssensors.1c02125 35138092 10.1021/acssensors.1c02125

[39] W. Zhu, T. Ye, S.J. Lee, H. Cui, S. Miao, X. Zhou, D. Shuai, L.G. Zhang, Enhanced neural stem cell functions in conductive annealed carbon nanofibrous scaffolds with electrical stimulation. Nanomedicine. **14**, 2485–2494 (2018). 10.1016/j.nano.2017.03.018 28552650 10.1016/j.nano.2017.03.018

[40] T. Altman, A. Ionescu, A. Ibraheem, D. Priesmann, T. Gradus-Pery, L. Farberov, G. Alexandra, N. Shelestovich, R. Dafinca, N. Shomron et al., Axonal TDP-43 condensates drive neuromuscular junction disruption through Inhibition of local synthesis of nuclear encoded mitochondrial proteins. Nat. Commun. **12**, 6914 (2021). 10.1038/s41467-021-27221-8 34824257 10.1038/s41467-021-27221-8 PMC8617040

[41] L. Heyburn, M.L. Hebron, J. Smith, C. Winston, J. Bechara, Z. Li, I. Lonskaya, M.P. Burns, B.T. Harris, C.E. Moussa, Tyrosine kinase Inhibition reverses TDP-43 effects on synaptic protein expression, astrocytic function and amino acid dis-homeostasis. J. Neurochem. **139**, 610–623 (2016). 10.1111/jnc.13763 27507246 10.1111/jnc.13763

[42] K. Imamura, Y. Izumi, H. Banno, R. Uozumi, S. Morita, N. Egawa, T. Ayaki, M. Nagai, K. Nishiyama, Y. Watanabe et al., Induced pluripotent stem cell-based drug repurposing for amyotrophic lateral sclerosis medicine (iDReAM) study: protocol for a phase I dose escalation study of bosutinib for amyotrophic lateral sclerosis patients. BMJ Open. **9**, e033131 (2019). 10.1136/bmjopen-2019-033131 31796494 10.1136/bmjopen-2019-033131 PMC7003406

