# Supplementary material for: Biohybrid motor neuron spheroid composed of graphene/HUVEC/neural cell for 3D biosensing system to evaluate drug of amyotrophic lateral sclerosis
Source: Nano Converg. 2025 Jun 26;12:29. doi: 10.1186/s40580-025-00495-0 (PMC12202271; doi:10.1186/s40580-025-00495-0)
Supplement: Supplementary file 1 — Supplementary Material 1 [file 40580_2025_495_MOESM1_ESM.docx]

Supporting Information

**Biohybrid motor neuron spheroid composed of neural cell/ graphene/HUVEC for 3D biosensing system to evaluate drug of amyotrophic lateral sclerosis**

*Minkyu Shin ^1,2,^*^†^*, Taehyeong Ha ^1,^*^†^*, Sangeun Lee ^1^, Jin-Ha Choi ^3,*^, Jeong-Woo Choi ^1,*^*

^1^ Department of Chemical & Biomolecular Engineering, Sogang University, Seoul 04170, Republic of Korea

^2^ Department of Chemistry and Chemical Biology, Rutgers, The State University of New Jersey,123 Bevier Road, Piscataway, NJ 08854, USA

^3^ School of Chemical Engineering, Jeonbuk National University, 567 Baekje-daero, Deokjin-gu, Jeonju-si, Jeollabuk-do 54896, Republic of Korea

^†^These authors equally contributed to this work

^*^Corresponding authors: Jeong-Woo Choi (jwchoi@sogang.ac.kr) and Jin-Ha Choi ([jhchoi@jbnu.ac.kr](mailto:jhchoi@jbnu.ac.kr))

**TABLE OF CONTENTS FOR SUPPORTING INFORMATION**

1. **EXPERIMENTAL DETAILS**
2. **SUPPLEMENTARY FIGURES**

**Fig. S1** Optical images of MNSs formed using round-bottomed ultra-low attachment 96-well plates by seeding the hNSCs with 0.1 mg/mL and 0.5 mg/mL of rGOps.

**Fig. S2** Generation and characterization of muscle bundle. (A) Schematic of fabrication and differentiation of muscle bundle. (B) Immunostaining images of muscle bundle cultured containing hydrogel for 7 days in differentiation media. (C) Contraction of muscle bundle upon electrical stimulation (1 Hz, 10 V).

**Fig. S3** Schematic of the co-culture process of muscle bundle and neural biohybrids.

**Fig. S4** Immunostaining images of Day 14 of NMJ differentiation with biohybrids MN spheroid. α-actinin (sarcomeric α-actinin, red), nAChR (α-BTX, White and neuron (Tuj1, green).

**Fig. S5** Confirmation of the effect of the rGOps and HUVEC on the biohybrid MN spheroid. (a) Optical image of the incorporation of rGOps within biohybrid cerebral organoid. (b) Cell viability assay *P < 0.05, **P < 0.01, and ***P < 0.001. Error bars correspond to the standard error of the mean from five measurements

**Fig. S6** Analysis of the electrophysiological signal of biohybrid cerebral organoid.

**Table S1** List of RT-qPCR primers

1. **SUPPLEMENTARY VIDEO CLIPS**

**Video S1** Spontaneous muscle contraction

**Video S2** The muscle bundle contractions following chemical stimulation

**A. Experimental details**

**Muscle bundle fabrication:** Muscle cells, C2C12 cells (ATCC, Gaithersburg, USA), were maintained in a growth medium consisting of DMEM with 10% FBS, 100 U/mL penicillin-G, and 100 μg/mL streptomycin; the growth medium was replaced every two days. C2C12 cells at a 500 × 10^4^ cells/mL density, 30% Matrigel, 4 mg/mL fibronectin, and thrombin (0.5 U/1 mg fibrinogen) were mixed to prepare the muscle cell hydrogels. The mixed hydrogels were distributed within the PDMS mold to fabricate muscle bundle, then differentiated in DMEM containing 2% horse serum, 1 mg/mL aminocaproic acid (ACA), 1 ng/mL insulin growth factor-1 (IGF-1), and the same antibiotics as the growth medium. The differentiation medium was replaced every two days for two weeks.

***qPCR:*** A reverse transcription polymerase chain reaction (RT-PCR) confirmed the ALS-biohybrid and NMJ differentiation. Genomic RNA was isolated from samples with a TRIzol reagent, and reverse transcription was performed using a SuperScript III kit (ThermoFisher, Waltham, USA). RT-PCR was completed with a FlexCycler2 system (Analytik Jena, Jena, Germany) using AccuPower Taq PCR PreMix (Bioneer, Oakland, USA). Lastly, glyceraldehyde-3-phosphate dehydrogenase (GAPDH—a housekeeping gene) mRNA expression levels were used to normalize all PCR experiments. The primers used are listed in **Table 1**.

**Electrophysiological activity analysis:** A multi-electrode array (MEA) was used to measure the neural biohybrid’s spontaneous electrophysiological activity, and MEA recordings were captured using a Maestro Edge system from Axion BioSystems (Atlanta, USA). MEA plates containing a 16-electrode array were first coated with laminin for 3 h at 37°C. Samples (neural biohybrids) were placed on an electrode array, and spontaneous electrophysiological activity was recorded in the culture medium (12.5 kHz for 5 min at 37°C). Axion Integrated Studio (AxlS 20.4.21) set a Butterworth passband filter with a 6 x SD threshold and a 200 to 3,000 Hz cut-off frequency as optimal conditions for minimizing false-positive signals.

**Immunostaining:** The neural hNSC biohybrid muscle bundle, made of iPSCs and ALS-iPSC-derived NSCs, and NMJ were fixed with 4% (w/v) paraformaldehyde (Sigma-Aldrich) for 1 hour and washed thrice with DPBS (WELGENE, Gyeongsangbuk-do, Republic of Korea). The fixed cells were then permeabilized with 0.2% (v/v) Triton X-100 (Wako, Osaka, Japan) for 1 hour, washed thrice with DPBS, and blocked with 1% (v/v) BSA (Sigma-Aldrich) for another hour. The samples were incubated overnight at 4°C with the following primary antibodies: mouse monoclonal anti-CD31 (1:500, Abcam, Cambridge, the United Kingdom), rabbit monoclonal anti-sarcomeric alpha-actinin (SAA) (1:1000, Abcam), rabbit monoclonal anti-neuron-specific class III β-tubulin (Tuj1) (1:1000, Abcam), mouse monoclonal anti-islet1 (islet1) (1:500, Abcam), Alexa Flour 488 alpha-bungarotoxin (α-BTX) (1:500, Invitrogen, ThermoFisher, Waltham, USA), and Alexa Flour 546 Phalloidin (F-action) (1:1000, Invitrogen). The samples were gently rinsed thrice with DPBS and incubated with secondary antibodies: Texas Red-conjugated goat anti-mouse IgG (Abcam) and Alexa Fluor 647-conjugated goat anti-rabbit IgG (Abcam). A confocal microscope (LSM 710, Carl Zeiss, Jena, Germany) was used to capture immunostaining images.

**Live/dead cell viability assay:** Next, a live/dead dye (1000X, Abcam) in DMSO was prepared to analyze cell viability. The live/dead dye was diluted to a 5X concentration with DBPS. Equal volumes of the neural biohybrid suspension and 5X concentration live/dead dye solution were mixed and incubated in the dark for 10 min at room temperature. Live/dead images of the neural biohybrids were obtained with a confocal microscope (Zeiss LSM710 microscope) and analyzed using ImageJ.

**B. SUPPLEMENTARY FIGURES AND TABLES:**


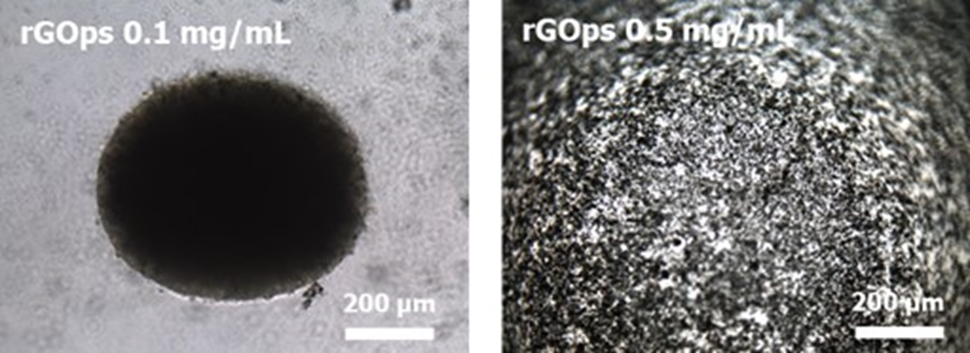


**Fig. S1. Optical images of MNSs by seeding the hNSCs with 0.1 mg/mL and 0.5 mg/mL of rGOps.**


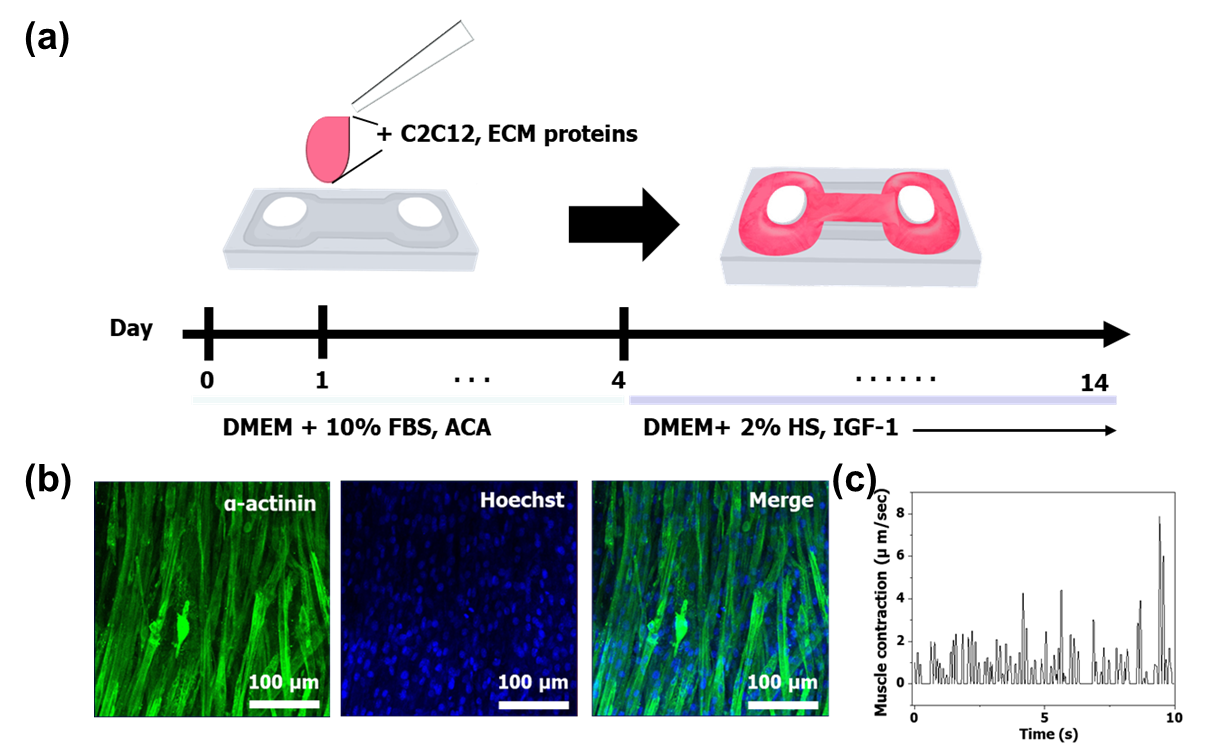


**Fig. S2. Generation and characterization of muscle bundle.** (a) Schematic of fabrication and differentiation of muscle bundle. (b) Immunostaining images of muscle bundle cultured containing hydrogel for 7 days in differentiation media. (c) Contraction of muscle bundle upon electrical stimulation (1 Hz, 10 V).


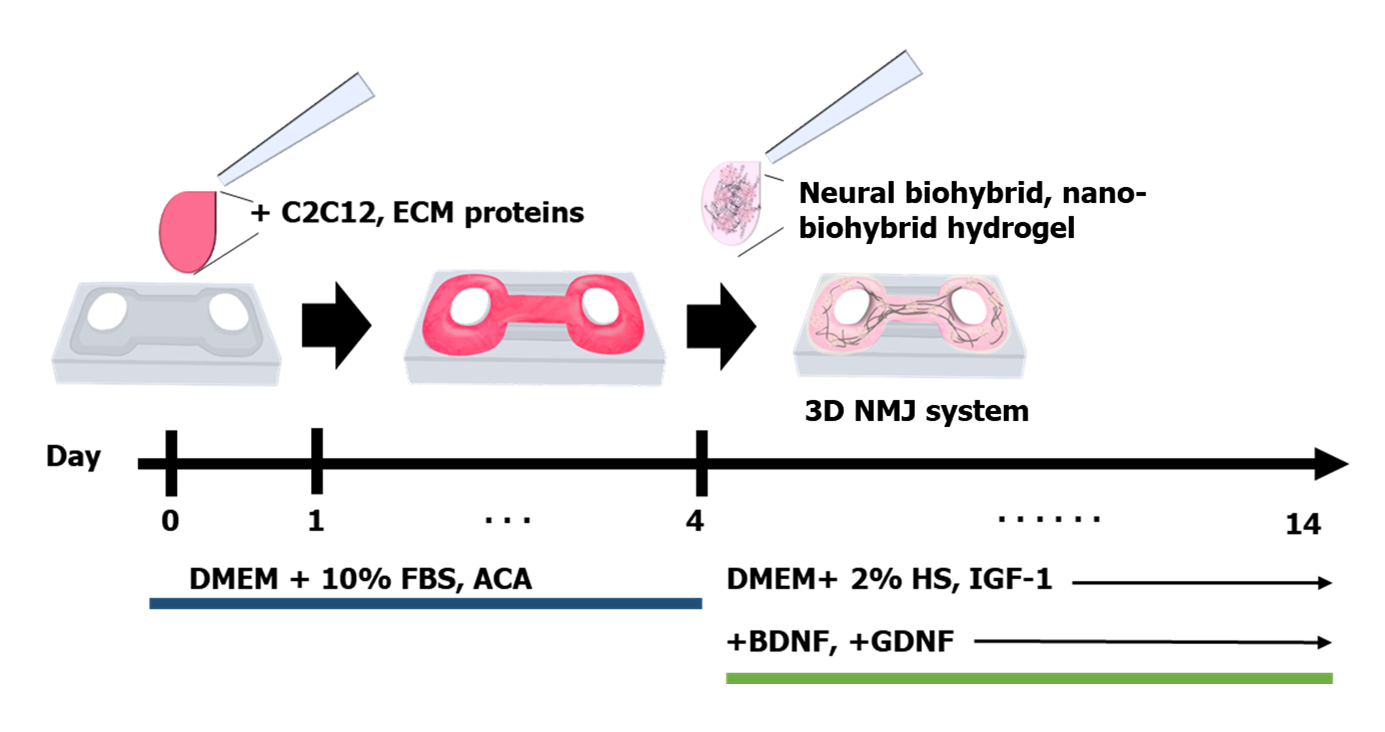


**Fig. S3.** **Schematic of the co-culture process of muscle bundle and neural biohybrids.**


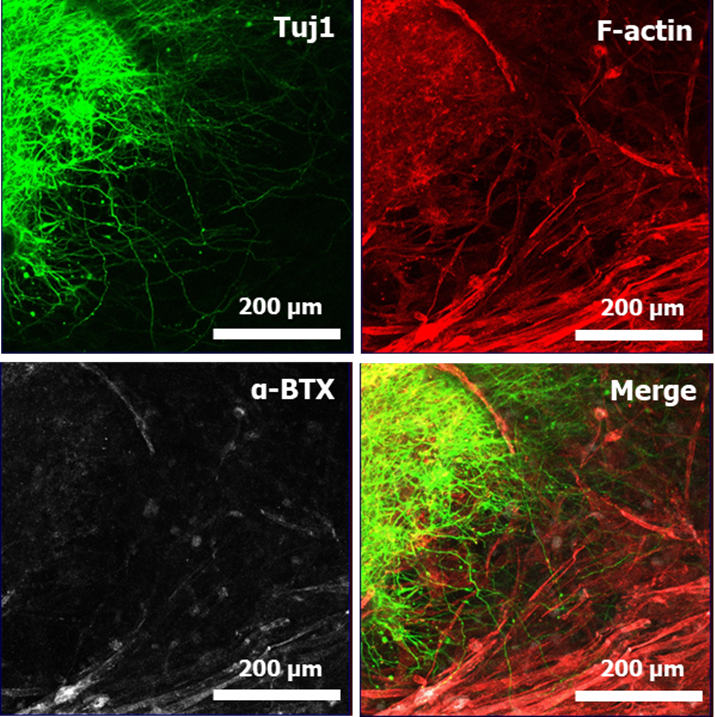


**Fig. S4.** **Immunostaining images of Day 14 of NMJ differentiation with biohybrids MN spheroid.** α-actinin (sarcomeric α-actinin, red), nAChR (α-BTX, White and neuron (Tuj1, green).


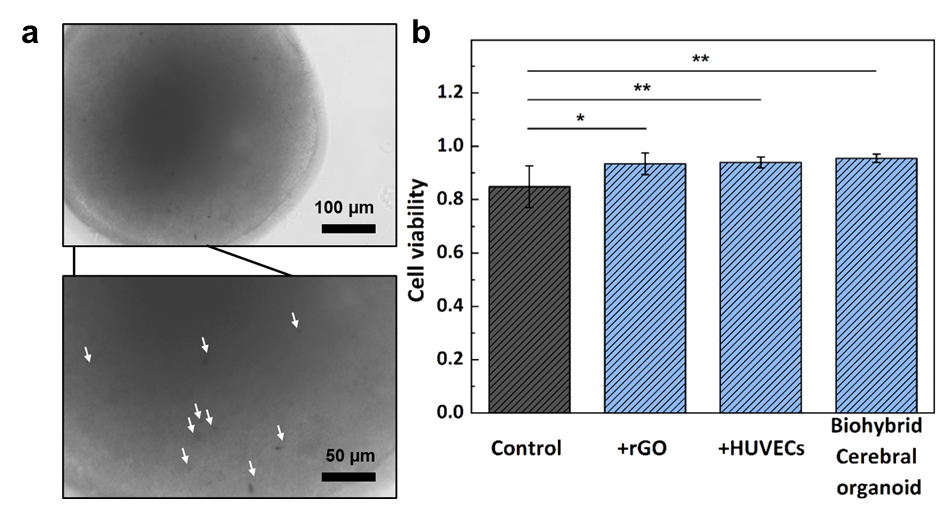


**Fig. S5.** **Confirmation of the effect of the rGOps and HUVEC on the biohybrid MN spheroid.** (a) Optical image of the incorporation of rGOps within biohybrid cerebral organoid. (b) Cell viability assay *P < 0.05, **P < 0.01, and ***P < 0.001. Error bars correspond to the standard error of the mean from five measurements


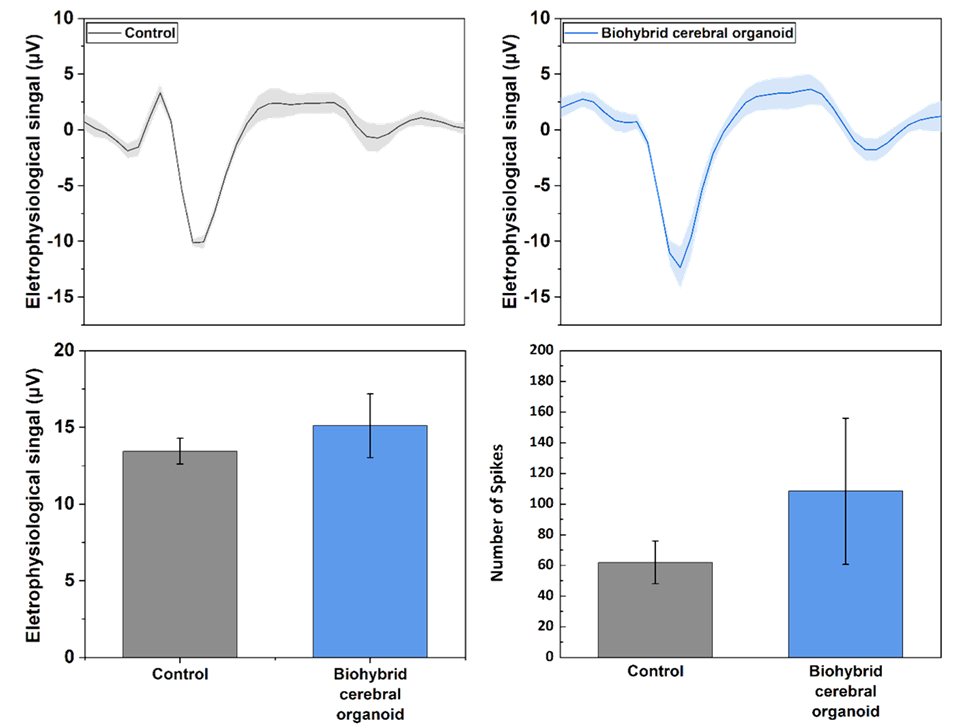


**Fig. S6.** **Analysis of the electrophysiological signal of biohybrid cerebral organoid.**

**Supplementary Table 1.** List of primers for qPCR

| **Gene** | **Forward Primers** | **Reverse Primers** |
| --- | --- | --- |
| *ChAT* | CCCACTAGCTGGAATCAGGC | GTACTGCCCATCCAGCCTTT |
| *SIM-32* | GCAGTCCGAGGAGTGGTTC | CGCATAGCGTCTGTGTTCA |
| *islet1* | TTGCTTAGCCTGTGCAGACA | TCCATCTGGGAGCTGACACT |
| *TDP43* | GCGCTGTACAGAGGACATGA | AGTTCATCCCACCACCCATA |
| *NEFL* | GAGTGGCTTTCGGCTTGCT | CACATTGCCGTAGATCCTGAA |
| *NEFM* | AAGGCAGTGGGAGGGAAGAG | TTCATCTGCTGGGCTCAAGTC |
| *CHRNA* | CAATGACTCGCAACCACTCA | GTGATCTGTCCAAGACATTTGC |
| *GAPDH* | CTGAGGCTCCCACCTTTCTC | AAGAGTTGTCAGGGCCCTTTT |
